# The Role of Magnetoencephalography in the Early Stages of Alzheimer’s Disease

**DOI:** 10.3389/fnins.2018.00572

**Published:** 2018-08-15

**Authors:** David López-Sanz, Noelia Serrano, Fernando Maestú

**Affiliations:** ^1^Laboratory of Cognitive and Computational Neuroscience (UCM-UPM), Centre for Biomedical Technology (CTB), Technical University of Madrid (UPM), Madrid, Spain; ^2^Department of Experimental Psychology, Complutense University of Madrid, Madrid, Spain; ^3^Biomedical Research Networking Center in Bioengineering, Biomaterials and Nanomedicine, Zaragoza, Spain

**Keywords:** magnetoencephalography (MEG), Alzheimer’s disease, mild cognitive impairment (MCI), subjective cognitive decline, preclinical Alzheimer’s disease

## Abstract

The ever increasing proportion of aged people in modern societies is leading to a substantial increase in the number of people affected by dementia, and Alzheimer’s Disease (AD) in particular, which is the most common cause for dementia. Throughout the course of the last decades several different compounds have been tested to stop or slow disease progression with limited success, which is giving rise to a strong interest toward the early stages of the disease. Alzheimer’s disease has an extended an insidious preclinical stage in which brain pathology accumulates slowly until clinical symptoms are observable in prodromal stages and in dementia. For this reason, the scientific community is focusing into investigating early signs of AD which could lead to the development of validated biomarkers. While some CSF and PET biomarkers have already been introduced in the clinical practice, the use of non-invasive measures of brain function as early biomarkers is still under investigation. However, the electrophysiological mechanisms and the early functional alterations underlying preclinical Alzheimer’s Disease is still scarcely studied. This work aims to briefly review the most relevant findings in the field of electrophysiological brain changes as measured by magnetoencephalography (MEG). MEG has proven its utility in some clinical areas. However, although its clinical relevance in dementia is still limited, a growing number of studies highlighted its sensitivity in these preclinical stages. Studies focusing on different analytical approaches will be reviewed. Furthermore, their potential applications to establish early diagnosis and determine subsequent progression to dementia are discussed.

## Introduction

Dementia is a condition usually affecting people aged older than 65 years that causes major cognitive dysfunction and loss of independence. Recent studies have demonstrated an incidence reduction over the past decades (Schrijvers et al., 2012; Matthews et al., 2013) associated to a reduction in exposure to dementia risk factors, such as improved management of cardiovascular risk factors, physical inactivity or smoking. However, this trend is not enough to compensate for the vast increase in longevity, and therefore the prevalence of people affected by dementia is expected to grow, increasing by a threefold by 2050. This picture turns dementia, and particularly Alzheimer’s Disease (AD) as it accounts for more than a 60% of dementia cases, into one of the main challenges for societies in 21st century. To this aim, a myriad of different compounds have been tested in AD patients, to stop or slow the progression of the disease yielding negative results in all the studies (Winblad et al., 2016). As a consequence, the focus in clinical trial design is shifting toward earlier stages of the disease, known as preclinical and prodromal AD (Livingston et al., 2017). Thus, its early identification is becoming increasingly relevant in current research literature, as it can help guiding targeted interventions and clinical trials.

Mild cognitive impairment (MCI) was first described in an attempt to fill the diagnostic gap between a healthy state without cognitive impairment and dementia, previously undiagnosed (Petersen et al., 2014). This intermediate stage is characterized by the presence of cognitive deficits with preserved independence. MCI is solidly conceived as a potential marker of AD pathology under certain conditions, and patients are known to be at a highly increased risk of dementia (Bruscoli and Lovestone, 2004). In the recent years, researchers have proposed that subjective cognitive decline (SCD) may represent an even earlier stage in the preclinical phases of AD (Jessen et al., 2014). SCD is characterized by a subjective feeling of cognitive worsening without objective neuropsychological deficits. Although very little literature have addressed the specific deficits in this population, multiple studies highlighted their relevance for subsequent cognitive deterioration (Tomaszewski Farias et al., 2017) and increased risk of AD (Wolfsgruber et al., 2016).

Current validated biomarkers for early AD detection include the presence of tau pathology (CSF or PET-Tau), amyloid pathology (CSF Aβ42 or PET-amyloid) and gray matter atrophy. However, as highlighted by the International Working Group, structural and metabolic changes, occurring many years before the onset of clinical symptoms, should induce functional changes which can be well captured with electrophysiological techniques (Dubois et al., 2016). In this vein, electroencephalography (EEG) and magnetoencephalography (MEG) offer a great temporal resolution to characterize subtle changes in brain dynamics non-invasively, which represent a major advantage in comparison with present biomarkers. A second advantage of MEG/EEG in the search of a biomarker is its ability to capture the magnetic field produced by intraneuronal currents, providing a more direct index of neuronal activity than methods relying on hemodynamic measures. Cortical synchronous activity is known to underlie normal cognition (Wang, 2010), which is heavily affected in dementia. Furthermore, electrophysiological measures have been recently proven useful in tracking brain activity disruption in relation to AD pathology, such as Aβ and tau deposits (Stoiljkovic et al., 2018). For these reasons, electrophysiological measurements represent a potential good candidate for AD detection. Furthermore, MEG is better suited than EEG as it allows exploring potential brain changes with a higher spatial resolution (Hedrich et al., 2017), increasing its ability to detect specific biomarkers, while offering a better signal-to-noise ratio in addition (De Jongh et al., 2005).

The aim of this work is to briefly summarize the state-of-the-art in the study of the preclinical and prodromal stages of AD with MEG. While specific findings regarding later stages of the disease, or from different neuroimaging modalities are briefly touched for the sake of contextualization, it is out of the scope of this work to summarize the vast literature in those fields (for a review on the use of MEG in AD see, Engels et al., 2017). This brief review is divided according to the different analytical methodologies employed in the studies. We reviewed results from different searches executed on PubMed, ScienceDirect and Google Scholar searching for the terms “preclinical Alzheimer” OR “mild cognitive impairment” OR “subjective cognitive decline” OR “subjective memory” AND “magnetoencephalography.” Titles and abstracts were screened to find original research articles using MEG and characterizing preclinical AD, a total of 48 articles were selected for review.

## Entropy and Complexity of the Brain Activity in the Early Stages of AD

These metrics assume that brain activity is generated by non-linear couplings between different neuronal populations. In general, these analyses estimate the predictability of brain signals to infer, in the case of complexity algorithms, the number of frequency components underlying the observed activity (Anokhin et al., 1996). There are a limited number of studies addressing complexity brain changes in the preclinical stages of AD. One of the first studies in the field reported a significant decrease in Lempel-Ziv complexity of MEG brain signals in AD patients as compared to controls (Fernández et al., 2010). More importantly, MCI patients showed intermediate complexity values between healthy elders and AD, although these differences were not significant. This tendency toward simpler brain activity implies an increase in signal predictability and is interpreted as a loss of independent oscillators underlying brain activity. In a similar vein, another study reported a highly significant decrease in spectral entropy in AD with MCI patients showing slight decreases over right lateral sensors, which was associated with a decrease in brain signal information content (Bruña et al., 2012). However, in this same study a significant increase in a disequilibrium measure, and a complexity metric based on Lopez Ruiz-Mancini-Calbet index (LMC) was reported for MCI patients compared to controls, and this was interpreted as possible compensatory mechanisms preceding a decrease in these parameters. These discrepant results are most likely due to the use of different metrics for complexity estimation. More recent studies addressing this topic seem to support previous results by using different metrics such as Jensen’s divergence and wavelet turbulence, reporting a decrease in complexity values from healthy to MCI and then to dementia stages (Poza et al., 2014a,b). Importantly, all studies were conducted at the sensor level, which represents a current limitation of the field.

## Activation and Power Analyses in the Early Stages of AD

Alzheimer’s Disease is widely known to affect power spectrum properties in the human resting state by increasing the relative contribution of slow frequency rhythms, and decreasing high frequency oscillations (Berendse et al., 2000; Huang et al., 2000; Fernández et al., 2002). Furthermore, this functional deterioration has been linked with hallmark structural abnormalities in AD such as hippocampal atrophy (Fernández et al., 2003). Interestingly, results in the MCI stage suggest a progressive deterioration of the synaptic activity in the early stages of the disease. For instance, López et al. (2014b) reported that MCI patients showed a similar pattern to that reported in AD with relative power increases in delta and theta band, and decreases in alpha and beta over posterior sensors. Interestingly, these changes were more accentuated in multi-domain MCI. In fact, these changes are able to track disease severity, distinguishing between healthy elders, MCI and AD patients (Besga et al., 2010; Fernández et al., 2013). In a recent study (Nakamura et al., 2018), delta power increases over fronto-medial cortex were related to entorhinal cortex shrinkage and reduced metabolism in the posterior cingulate cortex in MCI patients with positive amyloid-PET. Furthermore theta power increases were more evident in those participants with lower hippocampal volume and worse cognitive status. Early changes in low frequency oscillations have been related to deficits in cholinergic transmission particularly affecting the early stages of the disease and reducing its effect as the disease progresses (Poirier et al., 1995), which reinforces the relevance of studying the electrophysiological alterations in the prodromal stages of the disease.

Alpha band, which is the strongest resting state rhythm, is slowed in AD patients, even in the early onset form (Engels et al., 2016). Remarkably, this shift to the left of the power spectrum is already present in MCI patients (Garcés et al., 2013) and seems to progress toward later stages of the disease (Fernández et al., 2006a). Although the literature is quite consistent in this regard, a study from Osipova et al. (2006) failed to find a significant alpha slowing in the MCI group in the source space. However, their results might be hobbled by the small sample size in the MCI group, who exhibited a mean individual alpha peak 1 Hz slower in average compared to the control group. Interestingly, alpha reactivity to eyes-opening/eyes-closure seems to be maintained in MCI participants (Kurimoto et al., 2008).

Electrophysiological changes in the asymptomatic at-risk stages have been scarcely studied to date. However, a significant alteration of the spontaneous alpha activity is already present in SCD elders, who exhibited a decrease in alpha relative power at the source level despite not showing alpha peak slowing (López-Sanz et al., 2016). This alteration was related to a decrease in cognitive performance in the sample, thus emphasizing the relevance of subjective concerns as one of the first symptoms potentially revealing underlying brain pathology in the preclinical stages of AD. Alpha power reduction over posterior sources has been recently associated to gray matter loss in occipital regions in the AD-continuum (Babiloni et al., 2015). Although alpha power reduction has been an ubiquitous finding it has been recently found that amyloid depositions over prefrontal regions are associated to alpha power increases (Nakamura et al., 2018). Furthermore, Cuesta et al. (2015a) reported source-level power alterations also in healthy elderly carriers of the APOE 𝜀4 allele, who showed a significant increase of spontaneous theta power.

Regarding brain activity during task performance, some studies have reported an increase in brain activation in MCI patients (Püregger et al., 2003), and even in SCD (Maestu et al., 2011) while executing memory tasks. These findings were interpreted as a compensatory mechanism in the pathological group, who according to this hypothesis, would require additional activation in order to successfully perform the task. Nevertheless, there are also contradictory findings, since a subsequent study did not find any change in MCI source-level activity during a Sternberg-like memory task (Kurimoto et al., 2012).

There are a number of different toolboxes and packages able to compute these analyses such as Fieldtrip (Oostenveld et al., 2011), MNE Python (Gramfort et al., 2013), EEGLAB (Delorme and Makeig, 2004), or Brainstorm (Tadel et al., 2011) among others.

## Functional Connectivity in the Early the Stages of AD

Functional connectivity (FC) estimates the statistical dependence between two time series of brain activity. The evaluation of FC in brain imaging is motivated by the assumption that successful cognitive processing results from distributed and coordinated activity between distant brain regions. FC disruption in AD patients is a well-established finding in different neuroimaging modalities (Stam, 2010; Dennis and Thompson, 2014). However, a detailed description of FC trajectory in the early stages of AD could set the basis for the identification of a potential disease biomarker, as synaptic and functional deficits are known to appear earlier over the pathologic cascade than structural alterations (Jack et al., 2010).

Alzheimer’s Disease is considered a disconnection syndrome (Bokde et al., 2009), since FC is known to heavily decrease in dementia. Interestingly, sensor-space MEG studies have demonstrated that a significant decrease of mean FC is already present in the MCI stage in the beta band (Gomez et al., 2009). Further studies evidenced that temporal areas, supramarginal gyrus and right precentral showed decreased FC in the beta band in this population (Cuesta et al., 2015b). Furthermore, in the same study MCI patients also showed a significant disconnection in the alpha band, affecting bilateral inferior parietal and hippocampus, regions known to be critically affected in AD progression (Hock et al., 2000; La Joie et al., 2012). On top of that, alpha band hypo-synchronization in the source space has been reported to track disease severity in a study including patients with MCI and AD dementia (Ranasinghe et al., 2014). Interestingly, FC disruption in the default mode network (DMN), which has been extensively studied in AD patients (Jones et al., 2015), seems to be related to structural disconnection as reported by Garcés et al. (2014). Gómez et al. (2017), reported a widespread synchronization decrease affecting mainly beta band by using Granger Causality.

Interestingly, a significant number of studies also reported synchronization increases over certain areas in early AD. A recent study (López-Sanz et al., 2017a) showed that MCI and SCD participants exhibited a very similar pattern of alterations combining: hyper-synchronization over anterior brain regions (affecting the connection between cingulate gyrus, frontal regions and anterior temporal areas) and hypo-synchronization affecting more posterior areas (including parietal, medial temporal structures, and occipital regions). This result is consistent with other MEG studies reporting similar alterations with different FC metrics in MCI patients (López et al., 2017) and particularly, with the study conducted by Canuet et al. (2015) in which mixed FC increases and decreases at the source-level were found in MCI patients in relation to abnormal CSF biomarker levels (i.e., Aβ42 and phospho-tau).

Studies in even earlier stages (amyloid-PET positive healthy elders), highlight the existence of hyper-synchronization affecting posterior brain regions, such as the connections between precuneus and inferior parietal lobules (Nakamura et al., 2017). This increase in FC was related to the brain load of amyloid deposits. Furthermore, healthy controls carriers of the APOE 𝜀4 allele showed a significant FC increase affecting fronto-temporal connections (Cuesta et al., 2015b). These MEG results, and others coming from different neuroimaging modalities (Andrews-Hanna et al., 2007; Jones et al., 2011, 2015), simulation studies (de Haan et al., 2017) and animal models (Busche et al., 2008), have led to the hypothesis that the prevailing FC disconnection observed in demented patients is preceded by a phase of hyper-synchronization over the long course of preclinical AD, that could be detected by MEG as a potential biomarker of underlying AD pathology. In later stages, brain regions tend to get progressively disconnected, as confirmed also in other neuroimaging modalities (Jones et al., 2015).

Synchronization abnormalities in the preclinical stages during task performance have been scarcely characterized with MEG. While two studies reported overall increased FC in MCI, particularly over interhemispheric frontal sensors (Bajo et al., 2010; López et al., 2014c), another study found mixed FC increases and decreases, also at the sensor level, while performing a memory task (Bajo et al., 2012b). In the latter study, functional networks of SCD elders behaved similarly to controls, but with slightly lower FC values. The increases found in MCI synchronization were interpreted as an increase in resource demands to accomplish the task.

Some useful packages, already mentioned, to calculate FC in neurophysiological datasets are Brainstorm and Fieldtrip. Furthermore, HERMES is a dedicated toolbox for FC analyses (Niso et al., 2013).

## Topological Network Properties in the Early Stages of AD

Graph theory offers a promising framework to study how brain networks are organized in normal and pathological conditions. AD patients in particular, show a tendency toward random structure of these networks (Engels et al., 2017). Although very scarcely, MEG studies in MCI patients revealed a significant disorganization with decreased small world, clustering and transitivity and a more segregated hierarchical source-level brain structure as revealed by increased modularity during resting state (López-Sanz et al., 2017b), which is consistent with changes in AD. Interestingly, SCD elders showed similar trends in these parameters with intermediate values for most of them, particularly in theta and beta bands. These results highlight that MEG is able to capture network disorganization in the very early stages of the disease, even in the absence of objective impairment. Furthermore, it was found that clustering alterations, revealing connection abnormalities between neighboring neural populations, are related with fractional anisotropy decreases in white matter tracts in MCI patients (Pineda-Pardo et al., 2014b). This finding underlines that functional and structural network alterations might be connected in the early stages of the disease.

Network topology has been also studied in MCI during task performance. Overall, results reflect a tendency toward more random and less efficient networks in patients compared to controls in sensor analyses (Buldú et al., 2011). This study concluded that MCI networks are affected by a loss of balance between segregation and integration leading to an increase in long-range synchronization to overcome task demands, resulting in less efficient and less complex networks (Ahmadlou et al., 2014).

Graph theory studies have paid special attention to the evolution of those regions with a more central role in the network structure, the so-called hubs. Hubs are known to be particularly affected in AD patients (Yu et al., 2017). MEG studies have also found that hub reorganization occurs already in the MCI stage, being the more central sensors in healthy controls those showing higher alterations in MCI (Navas et al., 2015). Interestingly, similar results have been observed in resting state, as MCI patients showed decreases in centrality over posterior brain regions including parietal and occipital areas, and increases over prefrontal regions (López-Sanz et al., 2017b).

There is a variety of software freely available specifically devoted to network analyses, such as toolboxes for Matlab for dynamic networks (Sizemore and Bassett, 2017), Gephi (Bastian et al., 2009), Brain Connectivity Toolbox (Rubinov and Sporns, 2010), or BrainNet Viewer (Xia et al., 2013) among others.

## Meg as a Potential Biomarker in Early Stages of AD

Early electrophysiological alterations represent a sensitive biomarker to distinguish between MCI patients and controls (Pineda-Pardo et al., 2014a; Amezquita-Sanchez et al., 2016). Furthermore, FC features extracted from MEG activity have proven to be reliable across different MEG laboratories, as it was shown in a multicenter study (Maestú et al., 2015). In this work, sensor-level synchronization patterns of the participants coming from different MEG systems from several countries were blindly assigned using a previously trained algorithm into either MCI or control group, obtaining a high sensitivity. This was the first blind randomized study done with MEG, able to demonstrate reliability across MEG sites and high accuracy for unseen data. Strikingly, MEG has demonstrated great sensitivity to detect those subjects at a higher risk of AD progression. Increases in parietal delta activity were associated to a 350% increase in the probability of progressing to AD (Fernández et al., 2006b). Moreover, in other resting state study, increases in occipital lobe theta band power combination with structural and neuropsychological measures were able to correctly detect those MCI patients converting to AD in a 2-year follow up (López et al., 2016). Similarly, an increase in activation of the ventral and dorsal pathways while performing a memory task was associated to AD progression in MCI participants (Maestú et al., 2011).

In the same vein, FC metrics have demonstrated great utility in predicting AD risk. Hyper-synchronization over parieto-occipital regions during a memory task was related to AD progression in a follow-up study (Bajo et al., 2012a). Accordingly, an increased synchronization between anterior cingulate and different brain regions was found to be predictive of subsequent dementia in MCI patients (López et al., 2014a) which seems to be consistent with previous reports on abnormal protein distribution in preclinical AD, showing particularly high amyloid deposits in the cingulate cortex (Shukla and Bridges, 1999).

All the above-mentioned evidence endorse the potential relevance of MEG as an early biomarker of AD burden, highlighting its potential to predict future dementia. However, literature is still scarce in comparison with other neuroimaging modalities, hence, more studies with larger populations and validation of the potential biomarkers identified in different samples is a mandatory step in the coming future. Furthermore, no study to date carried a systematic comparison of the different metrics available and their respective ability to discriminate between healthy volunteers and patients, which should be a future step in the literature. Regardless, power analysis have the most abundant and consistent body of literature, probably partly due to their simplicity and relatively easy implementation. FC results are also a very promising tool for the coming future as a biomarker, since they could detect brain alterations potentially more specific than power alterations. Network and entropy analyses in AD count with a smaller and less consistent background to date, which could be due to the difficulty and complexity of the analyses leading to heterogeneous methodology implementation. There are still limitations for MEG as potential AD biomarker, such as the scarce number of MEG systems available worldwide, which limits its development. However, findings relating MEG alterations with current biomarkers such as CSF, amyloid-PET, brain atrophy, hypometabolism and genetic profiles, indicate its high sensitivity to reveal the functional impairment due to underlying AD pathology.

## Author Contributions

DL-S reviewed literature and wrote the paper. NS wrote the paper. FM designed the study and reviewed the manuscript.

## Conflict of Interest Statement

The authors declare that the research was conducted in the absence of any commercial or financial relationships that could be construed as a potential conflict of interest.
